# The Architecture of Relational Crisis: How To Study Diverging Perspectives

**DOI:** 10.1007/s12124-025-09939-y

**Published:** 2025-10-30

**Authors:** Yair Neuman

**Affiliations:** The Functor Lab Ben-Gurion University of the Negev, Beersheba, Israel

**Keywords:** Interpersonal crisis, Methodology, Perspective, Bakhtin, AI

## Abstract

Relational crises are difficult to understand, partially due to the lack of methodologies for studying small systems. For example, a small dynamic system such as a married couple is, on the one hand, irreducible to the psychology of its components and, on the other hand, too small for modeling through the statistics of large systems. To address the challenge of modeling a relational crisis, and by drawing on the work of Bakhtin, I present a methodology that exposes the diverging perspectives of a couple. The methodology is illustrated through a careful micro-genetic analysis of Bergman’s seminal drama “Scenes from a Marriage”.

## Why Size Matters

Relational crises are so common that one might mistake them for simple mechanical devices that went wrong. However, a deeper examination of relational crises makes us realize that the system is much more complex than we might have naively assumed. For example, Bergman’s seminal drama - “Scenes from a Marriage” (Bergman, 1973) - presents a couple, Johan and Marianne, and how their relationships *non-linearly* evolve over the years. At the beginning of the drama, they are interviewed for a women’s magazine and presented as epitomizing the ideal couple: a “happy,” loving, and “strong” couple characterized by “mutual understanding” (ibid. p. 14). It all changes when Johan admits to having an affair and leaves Marianne and their two daughters. The crisis deteriorates into a situation where all hell breaks loose, and one wonders what happened to this ideal couple.

How difficult is it to understand the crisis? In retrospect, everything can be explained by finding all sorts of reasons why the perfect couple collapsed. As storytellers by nature, we can always fit a narrative to the data (Bruner, 1987). In this context, we can retrospectively identify warning signs for the evolving crisis. Still, they have no predictive explanatory value. For instance, one possible explanation for the crisis is the personalities of Johan and Marianne. When presenting themselves at the interview, Johan describes himself through a long list of self-praise, such as being “extremely intelligent,” “sexy,” and a “great lover.” This self-presentation, although profoundly contextual as all personalities (Mischel, 2004), may be indicative of a narcissistic personality with an inflated sense of self. In contrast, Marianne presents herself only with respect to Johan (as a wife) and her daughters (as a mother). Her self-presentation may indicate a dependent, insecure, and introverted personality who defines herself only in relation to others. Is it possible to explain the crisis as resulting from the clash of narcissistic and dependent personalities? The answer is negative. The same personalities who admit falling in love and maintaining a happy family fall into a painful and violent crisis. The explanation cannot be found in the personalities of Johan and Marianne. Such a reductionist and retrospective explanatory move has been tried repeatedly, ignoring a basic fact realized by physics long ago: size matters. When the system’s size increases, the system’s behavior may change for a very simple reason: *interactions* (Neuman, 2021). The whole differs from the sum of its parts because non-linear interactions between the components result in an emerging behavior that cannot be reduced to the behavior of the composing components. Therefore, explaining the couple’s crisis through the components (i.e., Johan and Marianne) is a limited move. Similarly, explaining the couple’s behavior through some macro-level perspective, averaging human behavior is a limited explanatory move because of the same reductionist fallacy. This time, the fallacy results from top-down reductionism. Indeed, when trying to understand their situation, Johan and Marianne seem baffled and blame various causes, from the failure of the other (i.e., a bottom-up form of reductionism) to social structures (i.e., top-down reductionism). They even blame their parents. None of these explanations satisfies them or the audience watching the evolving drama.

Realizing that something is happening *in-between* the components of the system is the first necessary step toward a possible understanding of couples and relational crises. Unfortunately, we lack an appropriate toolkit to address the challenge of analyzing the behavior of a small system by looking in-between. This paper presents one possible approach for analyzing a relational crisis. The aim is to present this approach’s descriptive and explanatory power and illustrate it through “Scenes from a Marriage.” As I hope to show, the methodology exposes hidden layers of the relationship that may help us better understand the crisis experienced by the “ideal” couple and how it evolved. I first present some of Bakhtin’s ideas used to construct the methodology.

## Bakhtin on Wholes and Individuals

The idea that the whole is different from the sum of its parts naturally corresponds with Bakhtin’s epistemology (Bakhtin, 1990). However, to understand the whole, one should start with the specificity and uniqueness of the individual. As Bakhtin argues, this uniqueness results from the specific perspective one holds from one’s unique position. When I feel pain, no one can feel the same as I feel it, from the first-person perspective. You can understand my pain, sympathize with it, or empathize with it, but you can never experience it from the same perspective. This “axiom” of uniqueness states that we all have non-exchangeable perspectives. It also entails a fundamental asymmetry accompanying every human interaction: “When we gaze at each other, two different words are reflected in the pupils of our eyes.” (ibid., p. xxii). Paradoxically, these incompatible perspectives entail the promise of covering the blind spot necessarily accompanying our unique perspective. When we gaze at each other, each one of us has “an excess of vision” relative to the other” (ibid). The other, through her outside perspective, holds “an excess of vision” that, when used to mirror my perspective, can somehow provide me with a better wholistic perception of myself. In the case of a couple, the “architectonics” of the couple, as a whole composed of two parts, is not given as a static structure but dynamically worked out through perspectives.

Being is an event, explains Bakhtin, and this idea echoes in some modern conceptions of physics. For example, Rovelli (2019) explains that a kiss is not an entity but an event. It is a relational activity existing in time. In this relational context, the meaning of a crisis can be interpreted as a break in the architectonics of the couple. To understand this negatively loaded break in architectonics, we should recall Bakhtin’s famous phrase, “We are all unique but never alone” (ibid., xxvi). The break is evident when co-working perspectives stop functioning. In other words, we are all unique, partially blind, and have an excess of seeing with respect to the other. In relationships, we dynamically and recursively mingle with these three perspectival aspects. As we are never alone, our existence is grounded in the intricate and dynamic web of perspectives through which our selves are formed through the others who are recursively formed by us (Neuman, 2009). A crisis is evident when this perspectival dynamics does not function properly to satisfy the needs of the interlocutors.

The event of forming the whole can be specified by adopting Bakhtin’s metaphor of the individual as an author. The same as the author must adopt the hero’s perspective, so is the other, mutually covering our blind spot. This activity can be described as a *projection*. One must somehow project oneself onto the other to maintain a functioning couple. The second aspect of this process is the ability to see oneself from the perspective of the other. As beautifully explained by Bakhtin (1990, p. 25):

- I must empathize or project myself into this other human being … see the world within him … as *he* sees this world; I must put myself in his place and then, after returning to my place, “fill in” his horizon through the excess of seeing which opens out from this, my own, place outside him.

In this paper, I adopt Bakhtin’s ideas to describe the evolving crisis of a couple through the analysis of perspectives and projections. The proposed methodology generates a measure that aims to grasp the difficulty in projecting oneself, seeing the other, and through the other.

## Representing Perspectives

The paper focuses on how each character in the drama represents itself and the other. It aims to understand the divergence and convergence of perspectives. In other words, one way to understand the evolving crisis is through the perspective gap between Johan and Marianne. As the text is complex, one way to simplify the perspectives is to use a powerful idea underlying the most advanced Large Language Models (LLMs): Word embedding (Neuman et al., 2022). The idea of embedding is simple. The meaning of a sign, whether the first-person pronoun “I” or the sign “cat,” can be represented by its contextual lexical environment. For instance, the meaning of “ice cream” can be represented by the semantic field in which the word “ice cream” appears in textual corpora. These words (e.g., cone, truck, vanilla) and the strength with which they accompany “ice cream” form a *semantic field* that defines the sign’s meaning. In the advanced LLMs that exist today, the embeddings are used to generate a contextual sense of meaning that highly resembles the one proposed by Bakhtin (Holquist, 2003). The paper does not further discuss the contextual nature of current LLMs (Neuman, 2024).

In its simplest form, word embedding can be applied to representing the self and others. For instance, in the interview, Marianne describes herself as a mother and a wife. Johan describes himself with more descriptions, but let us choose two: successful and sexy. To compare their self-perspectives, we can use a vector, an array of numbers composed of the four descriptions: mother, wife, successful, and sexy. The vector represents the extent to which each word is “loaded” on the description of the character’s self. Using only 1 and 0 as values, Marianne’s self-presentation vector looks as follows:
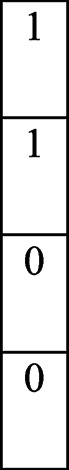


where the first cell corresponds to mother, the second to wife, the third to successful, and the fourth to sexy. In contrast, Johan’s self-perspective (i.e., Johan’s I) is represented as:
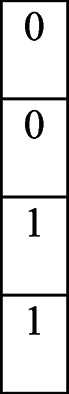


As we can see, the perspectives of Johan and Marianne are *orthogonal*, meaning they have nothing in common. Each person sees himself in a way that is incompatible with the self-perspective of the other. Therefore, projecting oneself onto the other is impossible. Johan and Marianne cannot see themselves through their partner because they share nothing in common.

The vector’s binary values I used above are simple but may be too simple. One may enrich the values by describing them on a continuous scale. To address this challenge, I first collected all the self and the other descriptions as they appear in the interview, opening the drama. To score the values, I present a general procedure illustrated with respect to Johan’s self-description. I used ChatGPT-4 and the following instructions:

- ◦ Here are several descriptions of a person: extremely intelligent, successful, youthful, well-balanced, sexy, concise, cultivated, well-read, popular, good mixer, friendly, likes sports, family man, good son, has no debts, pays taxes, respects the government, loves the royal family, and is a splendid lover.
- ◦ Imagine that you are a human being. You are exposed to n-1 of the descriptions and guess the likelihood of the missing description. Provide your answer on a scale of 0 (highly unlikely) to 1.

Using this prompt, ChatGPT scored each description. For example, “extremely intelligent” = 0.9, and “loves the royal family” = 0.5. As we would like to compare the self and other perspectives, I used all of the descriptions Marianne and Johan used and measured each description’s value. Next, I used ChatGPT to cluster the descriptions into semantic clusters by providing the following prompt:

- ◦ Cluster the following self-descriptions into six semantic clusters.

### The result was the following semantic clusters

#### Cluster 1: Intelligence and Success

- extremely intelligent.
- successful.
- cultivated.
- well-read.
- concise.
- has a natural appreciation for his excellence.

#### Cluster 2: Physical and Social Attractiveness

- youthful.
- sexy.
- popular.
- a splendid lover.
- has a nice figure.

#### Cluster 3: Social Skills and Friendliness

- good mixer.
- friendly.
- likes sport.
- rather nice.

#### Cluster 4: Family and Relationships

- family man.
- good son.
- mother.
- wife.

#### Cluster 5: Responsibility and Financial Stability

- has no debts.
- pays taxes.
- respects the government.
- well-balanced.

#### Cluster 6: Personality Traits

- loves the royal family.
- slow to anger.
- a fool.
- argumentative.

Next, I revised the vectors of Johan and Marriane as follows. Each vector is of length six, and each cell corresponds to a semantic cluster. The value of each cell is determined by the average score of the descriptions used for the person. For example, Marriane’s self-description is as a mother and a wife. Therefore, the first cell corresponding to *Intelligence and Success* scores 0. It expresses that none of her self-descriptions correspond with the semantic category of Intelligence and Success. Marianne does not seem to see herself in terms of intelligence and success. In contrast, Johan describes himself using five descriptions associated with the first cluster. The average score of these descriptions is 0.83; therefore, it is the first cell’s value in Johan’s vector. The vector representing Johan’s self-presentation is therefore:
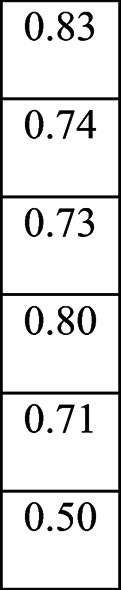


In sum, using the descriptions provided by each character, we can represent her/his self-perspective and her/his perspective of the other. This vectorial representation is sophisticated, as it represents the self and the other as points in a *high-dimensional semantic space* where the dimensions are the semantic clusters formed through the characters’ self-descriptions. The above example represents the self as a point or vector in a six-dimensional space. However, one can vary the dimensionality of this semantic space by increasing or decreasing the dimensionality. This form of representing the meaning of perspectives allows us to measure how the perspective of self (i.e., self-perspectives) and others diverge from each other and how differences and similarities of divergence evolve. The methodology I propose for measuring this divergence generates a single measure titled Vector Projection Divergence (VPD) (Neuman, 2025), and it is presented in the next section.

## Vector Projection Divergence (VPD)

This section describes the generation of the VPD. Readers who are not interested in the technical details may skip the section. However, they must understand that the VPD is an asymmetric measure of divergence between two vectors, such as those representing the self-perspectives of Johan and Marianne. In this case, we measure the divergence of Johan’s self-perspective (S_J_) from the self-perspective of Marianne (S_M_) and the divergence of Marianne’s self-perspective from the self-perspective of Johan.

Given two vectors, the procedure for computing the vector projection divergence is:

1.  Compute the dot product of v_2_ and v_1_.
2. Compute the dot product of v_1_ and v_1_.
3. Find the ratio between the first and the second result.
4. Multiply the value you produced in step 3 by v_1_.
5. Find the difference between v_2_ and the vector produced in step 4.
6. Find the length of the vector produced in step 5.
7. If you want to normalize the vector, divide it by the square root of n.

We start with two vectors, v_1_ and v_2_. To compute the *divergence of v*_*2*_ *from v*_*1*_, we first compute the projection of v_2_ onto v_1_. The projection is calculated as follows:

1. Projection of v_2_ onto v_1_:

$$Proj_{v1}{\boldsymbol V}_{\mathbf2}\boldsymbol\;=\frac{v2\cdot v1}{v1\cdot v1}V1$$

Where $\:{\text{v}}_{2}\cdot\:{\text{v}}_{1}$ It is the scalar product of the two vectors. The projection of v_2_ onto v_1_ represents the component of v_2_ that is in the same direction as v_1_. Next, we compute the projection error. This is the part of v_2_ not captured by v_1_:

2. Projection error:

$$Error_{v1}(\boldsymbol v\mathbf2)\:=\boldsymbol\:\boldsymbol v\mathbf2\;-\;Proj_{v1}\boldsymbol v\mathbf2\boldsymbol\;\;\;\;\;\;\;\;\;\;\;$$

The projection error is the difference between the original vector v_2_ and its projection onto v_1_. It represents the part of v_2_ that is not captured by the projection, showing how much v_2_ deviates from being in the direction of v_1_. Finally, we compute the

3. The divergence measure:

$${\mathrm D}_{v1}\left({\mathbf v}_2\right)=\left\|{\mathbf v}_2-{\mathrm{Proj}}_{v1}\;{\mathbf v}_2\right\|=\left\|{\mathbf v}_2-\frac{v2\cdot v1}{v1\cdot v2}v1\right\|$$

This measure represents how much of v_2_ is not aligned with v_1_ or how much v_2_ diverges from being a scaled version of v_1_. It is the magnitude (length) of the projection error vector and quantifies how different v_2_ is from v_1_ in terms of magnitude. It is an asymmetric and non-negative measure. To compare the divergence of vectors of different dimensions (e.g., the divergence of vectors with three dimensions to the divergence of vectors of six dimensions), we can normalize the divergence measure by dividing it by the square root of the vector length.

Let me illustrate the measure with respect to the two binary vectors we used to represent Marianne and Johan: M = [1, 1, 0, 0] and J = [0, 0, 1, 1]. Here is how we can calculate the measure:

Calculate the projection of J onto M:

The dot product J ⋅ M = (0 ⋅ 1) + (0 ⋅ 1) + (1 ⋅ 0) + (1 ⋅0) = 0.

The dot product M ⋅ M = (1 ⋅ 1) + (1 ⋅ 1) + (0 ⋅ 0) + (0 ⋅0) = 2.

Projection of J onto M:

$$\:Proj_JM=\frac{J\cdot M}{J\cdot M}M=\left(\frac02\right)\left[1,\:1,\:0,\:0\right]=\left[0,\:0,\:0,\:0\right]$$

The projection error is.

Error_M_(**J**) = **J** – Proj_M_**J =** [0, 0, 1, 1] – [0, 0, 0, 0] = [0, 0, 1, 1]

The final divergence measure is therefore:

$$\begin{array}{l}{\mathrm{VPD}}_{J\rightarrow M}=\left\|{\mathrm{Error}}_{\mathrm M}\left(\mathbf J\right)\right\|=\left\|\mathbf V\mathbf2-{\mathrm{Proj}}_{\mathrm v1}\mathbf v\mathbf2\right\|=\left\|\left[0,0,1,1\right]\right\|\\=\sqrt{0^2+0^2+1^2}=\sqrt2=1.414\end{array}$$

Where ‖**v**_**2**_- Proj_v1_**v**_**2**_‖ presents the norm (or length) of the vector formed by the error. Because the length of the vector is 4, the normalized divergence score of Johan from Marriane is approximately 0.707. In this case, the divergence score for both J→M and M → J is 0.414, meaning that the vectors are symmetric and orthogonal. This is a toy example, and I present the analysis of the real perspectives in the following sections.

## Johan and Marianne’s Diverging Perspectives

I calculated the VPD of Johan’s and Marianne’s self-perspectives. For brevity, they are described as S_J_ and S_M_. The divergence of S_J_ from that of Marianne (SJ → SM), and vice versa, may indicate how difficult it is for each of them to understand the other’s self-perspective through their self-perspective. Measuring the divergence between S_J_ and S_M_, we observe a clear asymmetry. Johan’s divergence from Marianne (S_J_ → S_M_) is higher than Marianne’s divergence from Johan by a factor of 2 (0.64 vs. 0.32, respectively). We may infer that it is much more difficult for Johan to understand Marianne by projecting his self-perspective onto hers. Following Bakhtin, this divergence of perspectives is inevitable. However, finding that the divergence is so powerfully asymmetric is less trivial. The measure indicates the relative efforts required by Johan to see Marianne. Paraphrasing Bakhtin, we can say that the efforts required by Johan to project himself into this other human being called Marianne are much higher than the efforts required by Marianne to empathize with Johan.

The second analysis compares each character’s self-presentation to how the other describes/conceives him. It is the gap between how one sees oneself and how the other sees oneself. For brevity, I use J_M_ and M_J_ to describe Johan’s description of Marianne and Marianne’s description of Johan.

The divergence S_J_ → M_J_ was higher than S_M_ → J_M_ (0.54 vs. 0.36, respectively). It means that the way Johan conceives and describes himself diverges from how Marianne sees him far more than the divergence between Marianne’s self-perspective and how Johan conceives her. In other words, there is also an asymmetry between how each character conceives himself and how his partner conceives him. Johan’s representation of his self diverges from how Marianne sees him. When looking at the mirror provided by the other, it is much more difficult for Johan to see himself.

It is important to emphasize that the divergence of perspectives indicates the dynamics between the partners. We do not simply compare or measure the distance between the perspectives of Johan and Marianne. The VPD is, by definition, relational and asymmetric. Therefore, it can expose a dynamic unseen through conventional interpretative or quantitative analysis. For example, each character presents itself in a way almost orthogonal to the other. This is clear to the attentive observer of the first scene. However, we do not easily see the *asymmetry* of their perspectives. The divergence of Johan’s self-presentation from Marianne’s self-presentation is significantly higher than the divergence of Marianne’s self-presentation from that of Johan. Moreover, the divergence between Johan’s self-presentation and how Marianne conceives him is significantly higher than between Marianne’s self-perspective and how Johan conceives her. This finding can be interpreted through Winnicott’s concept of “false self” (Winnicott, 2018). We will get to this point later.

What seems to be a simple gap between a highly self-assured and extroverted narcissist and his dependent and insecure wife may be enriched through the analysis. First, from Johan’s self-perspective centered around success, talent, and obedience, it is twice as challenging to see Marianne’s self, which is narrowed to being a wife and a mother. In simpler terms, it suggests that it is harder for Johan to align or “see” himself in the way Marianne sees herself. From a Bakhtinian perspective, there is an imbalance in how one can project oneself onto the other.

One may wonder whether these self-descriptions are contextual or indicative of a true self. Indeed, Johan’s self-presentation in the interview is contextual. He is deeply involved in impression management. However, how he presents himself is orthogonal to how Marianne presents herself in the *same context*. We do not adhere to some immanent or transcendental self when discussing true vs. false self. Our analysis contextualizes the false self as the divergence between one’s self-perspectives and how significant others conceive him. In Bakhtinian terms, the divergence may indicate a break in the activity of forming a whole.

Johan’s false self is exposed later in the drama. Although Johan presents himself as a family man, we later see that he expresses aggressive alienation towards their two daughters. Did he lie to the journalist when interviewed? Marianne reminds him of how caring he was with their daughters, as she is baffled by his uncaring and negative attitude. Who is the real Johan? The caring or the uncaring father? From a Bakhtinian approach, the false self is evident in the divergence of these behaviors and the inability of both Johan and Marianne to make sense of them. Johan’s relative difficulty in projecting himself onto Marianne or seeing himself through Marianne is, first and foremost, indicative of how alienated he is from him-self.

Moreover, the higher divergence between Johan’s self-perspective and the way Marianne conceives him shows that it is also more challenging for him to align his self-perception with the one provided by the outside perspective of his wife. Indeed, while Johan is the one initiating the separation, he is the one experiencing the most profound trauma, as seen in scene six. One explanation for this traumatic experience he initiated can be found in between his self-perspective and the outside perspective provided by Marianne. In Winnicott’s terms, Johan is unaware of his false self and the degree to which it diverges from his “true” self, as more accurately reflected by Marianne’s perspective.

## When all Hell Breaks Loose

The trajectory of the relationships hits bottom in scene six when all hell breaks loose. Measuring the VPD, I found that the divergence between S_J_ and S_M_ remains consistent (0.47 vs. 0.22, respectively), with Johan’s divergence two times higher than Marianne’s. This consistent divergence is interesting as it is expressed in their relationships’ highest and lowest points. It remains consistent across two diametrically opposing contexts. However, one may also notice that the divergence decreases for both. For Johan, from 0.64 to 0.47, and for Marianne, from 0.32 to 0.22. This decrease is explained by the fact that both share the same misery. Both “selves” are more symmetric in their misery but still preserve their asymmetric divergence.

In scene six, Johan is drunk, sick, and angry, but still finds it more difficult to see himself in the way Marianne sees herself. It seems that he learned nothing despite hitting rock bottom. However, the results are striking when examining the divergence of one’s self-perspective from the partner’s perspective. The divergence of S_J_ from M_J_ is far higher than that between S_M_ and J_M_ (0.42 vs. 0.04, respectively). The gap is ten times higher for Johan than for Marianne! In other words, the divergence between how Johan sees himself and how Marianne sees him is enormous. The divergence of perspectives has reached a peak when the situation deteriorates. One possible reason for this considerable asymmetry is that Marianne admits to being cheerful, as Johan bitterly and enviously describes. Both of them agree that she is cheerful. At the same time, she accepts Johan’s accusation, taking the blame for her “filthy temper.” In addition, the gap between how Marianne conceives herself and how Johan conceives her has been reduced enormously from the first scene to the sixth scene (0.36 to 0.04). This finding is not indicative of Johan’s improved mentalization. Instead, it is a process attributed to Johan’s shift from a “humorous” positive and shallow description of Marianne (e.g., nice figure) to a vicious and hostile description (e.g., worse than a whore), much aligned with Marianne’s negative aspects shared with Johan. It is the misery that aligns the perspective rather than an improved understanding.

Marianne is a much more complicated character than Johan. While Johan appears sick, drunk, depressed, and self-pitying, Marianne arrives cheerfully, admitting she is in love and finally free from Johan. At the same time, she acknowledges her painful parts while aligning with Johan’s negative description of himself by calling him crazy, cowardly, and childish.

Although the relationship has changed, the way Johan and Marianne see themselves in and through the other remains the same. This consistency is explainable through perspectives popping up along the process. One of these perspectives appears at the beginning of the play, where Marianne meets an old lady, unintentionally providing her with mirroring and reflection. This meeting adds another layer to the micro-genetic analysis of the evolving crisis.

## Mirroring Through an Old Lady

In scene two, Marianne, a lawyer dealing with divorce, is meeting with a new client, an old lady by the name of Mrs. Jacobi. Mrs. Jacobi expresses her wish to divorce her husband. When asked why she wants to divorce, she answers: “It is a loveless marriage.” (Bergman, 1973, p. 53). Her answer seems to surprise Marianne. This surprise is in itself surprising. As an audience, we do not understand why an experienced divorce lawyer should be surprised by a client’s justification for divorce. Marianne’s surprise seems to increase when Mrs. Jacobi describes her husband as kind, conscientious, and an excellent father. Although he is “very dependable,” they never quarrel, have no financial issues, and share their interest in music by playing chamber music together. Marriane describes this situation as *ideal*. Here, a linkage is formed between the “ideal” marriage of Marriana and the “ideal” marriage of Mrs. Jacobi. The only difference, if there is one, is a loveless marriage. This description leaves no doubt about Mrs. Jacobi’s reason for divorce. Her loveless marriage is the *only* reason for getting a divorce.

Marianne and Mrs. Jacobi’s husband do not understand the meaning of lovelessness; Marianne asks Mrs. Jacobi to explain what “form” lovelessness takes, while the husband asks Mrs. Jacobi to explain what love she seeks. An equivalence is formed between Marianne and Mr. Jacobi. In both cases, they seem to misunderstand the meaning of romantic love and its lack.

After admitting she never loved her children, although she was a good mother, Mrs. Jacobi turns to Marianne, saying she knows what she thinks of her. Marianne is quick to deny it. However, Mrs. Jacobi correctly guesses that Marianne conceives of her as a spoiled woman who has everything but still seeks something vague called love. Marianne then admits that “perhaps” she was thinking something of this kind. This confession indicates that Marianne behaves unprofessionally when judging her client. She becomes emotionally involved.

Mrs. Jacobi describes a life of depression where everything gets meaner and grayer. Her loveless marriage is “frightening” as it drains life out of her. Asked if her husband understands her, Mrs. Jacobi portrays a sad picture of her husband, who becomes bitter and bad-tempered whenever discussing the issue of divorce, calling her romantic and silly and explaining her wish to divorce by attributing it to a life crisis rather than acknowledging and respecting her. At this point, something suddenly arises in Marianne, who jumps to her feet to call Johan. The meeting with Mrs. Jacobi is suddenly terminated, and we understand that deep anxiety has been evoked in Marianne. This is an important turning point in the evolution of relationships.

I used the descriptions of Johan and Mr. Jacobi, and their automatic clustering resulted in three clusters: (1) Positive personality traits, (2) Negative personality traits, and (3) other complex traits. As a side note, one may realize that the semantic clusters indicate the contextual aspect of the personality. Mr. Jacobi is described as a kind person. However, when discussing divorce, he turns bitter and has a bad temper. The positive and negative aspects of Mr. Jacobi appear separated by two semantic clusters corresponding to two different contexts. This is probably why Mrs. Jacobi also describes him as “very dependable.” This understanding explains why the kind Mr. Jacobi describes his wife as romantic, silly, and suffering from a life crisis. He cannot let her go free.

I formed two vectors of length three for Mr. Jacobi = [0.8, 0.2, 0.7] and Johan = [0.8, 0, 0.7]. Using the VPD on the descriptions of Mr. Jacobi and Johan, it was found that the divergence of Mr. Jacobi from Johan is 0.12, and of Johan from Mr. Jacobi is 0.11. *Their description by their wives is similar to the level of symmetry*. This is something not explicitly discussed in the drama. From an artistic perspective, Johan and Mr. Jacobi are the same and psychologically interchangeable.

As we learned from Matte-Blanco’s insightful work (Matte-Blanco, 2003), symmetrization is the constituting logic of the unconscious. The symmetry of the VPD measure exposes the symmetry of Mr. Jacobi and Johan. At this point, we may better understand the turning point in the plot and why Marianne is so moved by a meeting that is supposed to be an ordinary professional meeting between a lawyer and her client. If Johan is the same as Mr. Jacobi, then maybe Marianne is similar to Mrs. Jacobi. The unconscious analogy may signal to Marianne that, similarly to Mrs. Jacobi, she also experiences a loveless marriage where she is depressed and drained of life.

How about the way Marianne describes herself versus the self-description of Mrs. Jacobi? Automatically clustering their descriptions resulted in three semantic clusters: (1) Familial roles, (2) emotional traits, and (3) complex and abstract traits. The vectors were Mrs. Jacobi = [0.7, 0.6, 0.5] and Marianne = [0.7, 0, 0]. The divergence of Marianne from Mrs. Jacobi was lower than that of Mrs. Jacobi from Marianne (0.30 vs. 0.45, respectively). It is smaller by a factor of 1.5. This finding can be interpreted as follows: Marianne can see herself in Mrs. Jacobi much more than Mrs. Jacobi can see herself in the lawyer, as both of them are mothers and wives (first cluster). However, Mrs. Jacobi can be less aligned with Marianne because Marianne’s description (unknown to Mrs. Jacobi) is drained of emotional traits. When describing herself in the opening scene, Marianne presents herself as a mother and a wife. No more. It is now clear why she thinks Mrs. Jacobi is “spoiled.” On the one hand, we have a woman who defines herself through her familial duties (as a wife and a mother). A woman who, when asked about love in the interview, dismisses all notions of romantic love; on the other hand, we have an old lady striving for love and ready to pay the price for being honest with herself. For Mrs. Jacobi, there is no false self. She courageously knows who she is and what she wants.

However, here is an interesting point. If Mr. Jacobi and Johan are unconsciously symmetric, then by inference, so are Mrs. Jacobi and Marianne. However, while Mrs. Jacobi describes herself as both depressed and having the capacity to love, both of these aspects are missing from Marianne’s self-description. Marianne’s perspective of herself is shallow, functional, and devoid of emotions. However, if Mr. Jacobi is symmetric with Johan, and Marianne can see herself in Mrs. Jacobi, can it be that the missing parts necessary for establishing a complete symmetry between Marianne and Mrs. Jacobi are those painful parts Marianne refuses to admit? Are these the same parts raising the anxiety to the level where the meeting is suddenly terminated?

**Fig. 1:**
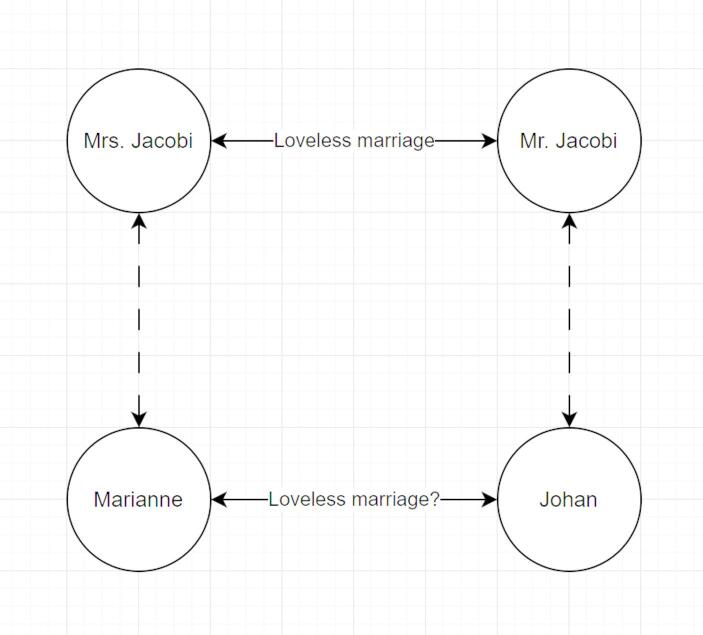
The emerging construct of relationships

This turning point in the drama is where Marianne becomes “unconsciously aware” of a hidden depression and loveless marriage. Something suddenly changes through the mirroring of the old lady and her perspective on her husband. Using the VPD, I identified a symmetry between Johan and Mr. Jacobi and Marianne and Mrs. Jacobi. The symmetry between Marianne and Mrs. Jacobi is not trivially exposed. They are similar in terms of their familial roles but different in terms of emotions. However, these differences lead to a strong emotional response in Marianne, indicating that maybe these differences are unacknowledged similarities. These perspectives and their hypothetical implied meaning for Marianne are presented in the following figure (Fig 1).

The event of forming a whole couple is challenged by the meeting with the old lady. Although Johan has not informed her yet about his affair, Marianne starts feeling that something is wrong. Her blind spot has been exposed through the excess vision provided by the old lady. Seeing herself both in Mr. and Mrs. Jacobi gives Marianne the excess of vision that evokes anxiety. She now understands better. Johan cannot project himself into Marianne’s uniqueness to the same extent as Marianne can project herself into Johan. Although they are unique, they are alone and, as such, cannot maintain a functioning whole that is challenged by evolving perspectives and circumstances.

## Discussion

This paper has presented a novel approach to analyzing relational crises, drawing upon Bakhtin’s dialogical framework and using the VPD to capture the diverging perspectives. Through a micro-genetic analysis of “Scenes from a Marriage,” I have demonstrated how the VPD can illuminate the subtle yet critical shifts in Johan and Marianne’s relationship, revealing asymmetries and divergences that conventional analyses might overlook. The analysis addresses the complexity of the couple’s dynamics. Using a methodology that is, by definition, relational, I avoided the two horns of the reductionist dilemma. Instead of analyzing the components, Johan and Marianne, or the social forces acting on them, I focused on the dynamics “in between.” More specifically, I looked at the degree to which Johan and Marianne can project their self-perspectives onto each other and perceive themselves through the other’s lens. The observed asymmetries suggest a fundamental imbalance in their relational architecture. This paper offers a methodological framework for understanding relational crises as a clash of perspectives quantified through the VPD. This approach, grounded in Bakhtin’s dialogical ideas, provides a nuanced and dynamic understanding of how relationships evolve and deteriorate. It emphasizes the importance of considering the “in-between” and the relational dynamics that shape individual experience. The methodology by no means pretends to be exhaustive. It does not provide THE “key” to the relational crisis experienced by a couple. Crises are multifaceted and, as such, require a toolkit rather than a single key. By providing a quantitative lens through which to examine the complexities of a relational crisis, the methodology provided one possible tool for understanding the intricate dance of relationships. We are all unique but never alone, but crises may sometimes remind us how lonely we are.

## Data Availability

No datasets were generated or analysed during the current study.

## References

[CR1] Bakhtin, M. (1990). *Art and answerability*. TX: University of Texas Press.

[CR2] Bergman, I. (1973). *Scenes from a marriage. *N.Y.: Bantam Books.

[CR3] Bruner, J. (1987). Life as narrative. *Social Research, *54, 11–32.

[CR4] Holquist, M. (2003). *Dialogism: Bakhtin and his world. *N.Y.: Routledge.

[CR5] Matte-Blanco, I. (2003). *Thinking, feeling, and being. *London: Routledge.

[CR6] Mischel, W. (2004). Toward an integrative science of the person. *Annual Review of Psychology,**55*(1), 1–22. 14744208 10.1146/annurev.psych.55.042902.130709

[CR7] Neuman, Y. (2009). Peter pan’s shadow and the relational matrix of the I. *Semiotica*, *176*, 15–27. 10.1515/semi.2009.058

[CR8] Neuman, Y. (2021). *How small social systems work: From soccer teams to jazz trios and families.* N.Y.: Springer Nature.

[CR9] Neuman, Y. (2024). *AI for Understanding context.* N.Y.: Springer.

[CR10] Neuman, Y. (2025). *AI for Understanding human conversations. *N.Y.: CRC/Routledge.

[CR11] Neuman, Y., Danesi, M., & Vilenchik, D. (2022).*Using AI for dialoging with texts: From psychology to cinema and literature.*N.Y.Routledge.

[CR12] Rovelli, C. (2019). *The order of time.* UK: Penguin.

[CR13] Winnicott, D. W. (2018). *The maturational process and the facilitating environment. *Routledge.

